# A Novel Variant of ARID1B-Related Coffin-Siris Syndrome in a Saudi Girl: A Case Report

**DOI:** 10.7759/cureus.88507

**Published:** 2025-07-22

**Authors:** Ali S Alquraishi, Syed Rayees, Musa M Saad, Hind Alasmari

**Affiliations:** 1 Department of Pediatrics, Armed Forces Hospital Southern Region, Khamis Mushait, SAU

**Keywords:** coffin-siris syndrome, developmental pediatrics, dysmorphism, hypertrichosis, novel variant

## Abstract

Coffin-Siris syndrome (CSS) is a rare genetic disorder characterized by underdeveloped toenails or fingernails, developmental delays, and intellectual disability, among other characteristics. The clinical manifestations can vary significantly. We present a case involving a Saudi girl who had global developmental delay, hypertrichosis, and subtle dysmorphic traits since birth but remained undiagnosed until the age of nine. A novel pathogenic heterozygous variant c.1314del p.(Glu439Serfs*63) in the ARID1B gene was identified through whole-exome sequencing (WES). Consequently, she was referred to a multidisciplinary team that managed her symptomatically. This case exemplifies the necessity of considering the syndrome in patients exhibiting developmental delays and unusual features such as hypertrichosis. Furthermore, this study highlights the importance of utilizing advanced medical technologies, such as WES, for timely diagnosis and effective management.

## Introduction

Coffin-Siris syndrome (CSS) is a rare genetic disorder that leads to developmental delays, intellectual disabilities, distinct facial features, hypertrichosis, sparse hair on the scalp, and either hypoplastic or absent fifth fingernails or toenails. Additional variable traits may include insufficient overall growth, hypotonia, craniofacial anomalies, spinal deformities, and congenital heart defects [1,2]. Hypertrichosis is characterized by an abnormal increase in hair growth in regions that normally do not exhibit thick hair coverage, and it is unusual for the person's age, gender, and ethnicity. CSS can be passed down as an autosomal dominant trait or arise from a de novo mutation. The ARID1B gene mutation is the most frequently observed genetic alteration associated with CSS and is characterized by a high degree of heterogeneity in the genotype-phenotype relationship [3-5]. Diagnosis generally depends on recognizing major clinical signs along with at least one minor sign, and it can be confirmed through molecular genetic testing of the implicated genes. Treatment mainly aims to provide support and mitigate symptoms, with recommendations for occupational, physical, and speech therapy [6-8]. The prognosis for patients with CSS remains largely unclear due to the absence of long-term studies. However, some study cohorts have found adult patients with CSS aged between 18 and 69 [4]. It is crucial to monitor development and feeding closely, and patients should undergo regular ophthalmological and audiological evaluations [7-9]. In this report, we discuss a case of CSS diagnosed at the age of nine, prompted by a complaint of hypertrichosis. The patient exhibited subtle dysmorphic features and developmental delays from infancy, yet the condition was not diagnosed earlier. This case highlights the critical role of genetic testing in patients presenting with global developmental delays (GDDs) and dysmorphic features, as an early diagnosis can facilitate appropriate management for the child.

## Case presentation

We present a case of a nine-year-old girl who was referred from the dermatology department to the endocrinology clinic, with the primary complaint of hypertrichosis. Upon thorough systemic examination, she displayed subtle dysmorphic features, characterized by a broad forehead and coarse facial attributes, in addition to generalized hypertrichosis. She was classified as Tanner stage 1, with normal genitalia. There were no signs of acne or baldness. The chest examination revealed clear breath sounds, a normal cardiovascular system with regular heart rhythms, and a soft, non-tender abdomen. A 2D echo showed no abnormality. Notably, she displayed global developmental delay along with indications of intellectual disability. Her weight was recorded in the 50th-75th percentile, while her height fell within the 25th-50th percentile range. She was born full term through spontaneous vaginal delivery, with no need for NICU admission, and she was not on any chronic medications. Her parents noted that her developmental milestones had been delayed since birth across all domains. There was no family history of a similar condition. At the age of four, she had presented to the emergency room with a complaint of loss of consciousness. An MRI of the brain conducted at that time yielded unremarkable results. Her parents had reported hypertrichosis and developmental delay during that visit. Unfortunately, no further investigations were pursued at that time, and she ceased attending follow-up appointments.

In the endocrinology clinic, she underwent a comprehensive investigation to ascertain the cause of her hypertrichosis. The blood workup performed was unremarkable (Table 1), confirming normal adrenal function and the absence of hyperandrogenism. A pelvic ultrasound also returned normal results. Given the normal routine blood workup and the presence of dysmorphic features, hypertrichosis, along with GDD, a whole-exome sequencing (WES) study was conducted (Table 2), which identified the pathogenic heterozygous novel variant c.1314del p.(Glu439Serfs*63) in the ARID1B gene, confirming a diagnosis of Coffin-Siris syndrome. She was subsequently referred to a multidisciplinary team comprising an endocrinologist, dermatologist, geneticist, neurologist, developmental pediatrician, and speech and language therapist for management and future follow-up.

**Table 1:** Blood workup

Description	Result	Reference range
White blood cell	4.9 x10^9^/l	4.5-13.5 x10^9^/l
Hemoglobin	13 g/dl	10.9-15 g/dl
Follicle-stimulating hormone	1.3 mlU/ml	0.91-7.8 mIU/ml
Luteinizing hormone	0.1 mlU/ml	Mid-follicular 2.12-10.89 mlU/ml, mid-cycle peak 19-103 mlU/ml, mid-luteal phase 1.20-12.86 mlU/ml
Prolactin	3.21 ng/ml	3.21-18.46 ng/ml
Thyroid-stimulating hormone	2.43 uIU/ml	0.79-5.85 uIU/ml
Free T4	13.13pmol/l	7.85-13.6 pmol/l
Testosterone	1.60 nmol/l	0.35-1.63 nmol/l
Dehydroepiandrosterone	2.6 U/l	0.43-5.6 U/l
Cortisol	350 nmol/l	60-353 nmol/l
17-hydroxyprogesterone	1.2 ng/ml	Upto 1.1
Androstenedione	0.53 ng/ml	No reference range known for this age

**Table 2:** Whole-exome sequencing study MIM: mendelian inheritance in man, AD: autosomal dominant

Gene (isoform)	Phenotype MIM number (mode of inheritance)	Variant	Zygosity	Classification
ARID1B	135900 (AD)	c.1314del p.(Glu439Serfs*63) chr6:157100373	Heterozygous	Pathogenic

## Discussion

Coffin-Siris syndrome is a rare genetic syndrome caused by mutations in the genes encoding subunits of the SWI/SNF complex, also known as the BAF (Brg-1-associated factors) complex, which plays a crucial role in regulating gene expression during development. BAF functions as an epigenetic regulator by changing the structure of chromatin, which allows transcription factors easier access to DNA. Several genes are known to be causative for CSS, including ARID1A, ARID1B, SMARCA4, SMARCB1, DPF2, SMARCE1, ARID2, SMARCC2, SOX11, SOX4, SMARCD1, and BICRA. Nicolaides-Baraitser syndrome (NCBRS) has a similar phenotype to CSS and can be kept as a differential diagnosis. CSS was first reported and discovered by Evelyn Siris and Grange S. Coffin in the year 1970 [1-2]. ARID1B-related disorder (ARID1B-RD) results from pathogenic heterozygous variants in the ARID1B gene, which can manifest as classic CSS and intellectual disability, with or without nonspecific dysmorphic features. The estimated incidence of this disorder ranges from 1 in 10,000 to 1 in 100,000 [3]. There are ongoing clinical trials investigating treatments targeting SMARCA4-associated tumors.

Pathogenic variants in the ARID1B gene on chromosome 6q25 are identified as the leading cause of CSS and are also among the most prevalent causes of intellectual disability. The range of phenotypes caused by ARID1B mutations is now considered to be extremely wide. CSS represents a disorder that is both clinically and genetically diverse, characterized by variable expressivity and incomplete penetrance. Although the exact prevalence is unknown due to the rarity of the syndrome and the limited knowledge of clinicians, fewer than 250 cases have been reported worldwide [2-4]. A wide range of clinical characteristics is linked to CSS, which encompasses a spectrum of both major and minor clinical findings. The primary major characteristics consist of developmental or cognitive delays ranging from mild to severe (present in all patients), hypoplasia or aplasia of the fifth fingernail/distal phalanx, hypertrichosis, and coarse facial features (typically noted over time). Notable facial traits include prominent eyebrows and lengthy eyelashes, a broad nasal bridge, a wide mouth with thick, everted upper and lower lips, and irregular ear positioning or shape. Additional major findings comprise short stature, failure to thrive, difficulties with feeding, microcephaly, ophthalmological issues (such as cataracts, ptosis, and strabismus), cardiac defects (including ventricular septal and atrial septal defects, tetralogy of Fallot, and patent ductus arteriosus), hypertrichosis (on the arms, face, and back), and sparse hair on the scalp. Minor findings may include neurological issues (such as Dandy-Walker malformation, gyral simplification, agenesis of the corpus callosum, seizures, and hypotonia), hearing impairment, joint laxity, genitourinary and renal anomalies, and recurrent infections. Developmental delays and scoliosis are seen mainly during infancy and childhood [5-6]. Patients with SMARCB1 mutation exhibit the most pronounced physical phenotype along with significant cognitive and growth delays. The variation in phenotype appears to be particularly significant in patients with mutations in ARID1A and ARID1B. Anomalies in the distal limbs are most evident in ARID1A patients, while they are least pronounced in those with SMARCB1 [6-8].

Diagnosis typically relies on recognizing clinical signs and symptoms, which are then validated through molecular genetic tests such as WES. Treatment primarily focuses on providing supportive care to address the patient's presentation [8-10]. At each follow-up, evaluation of growth and developmental progress and nutritional status is essential, including an annual ophthalmological assessment and a scoliosis evaluation until growth has concluded. Additionally, audiological assessments, dental assessment, behavioral evaluations, and hormonal evaluations/bone age assessments should be conducted as necessary, depending on the symptoms presented. Individuals experiencing seizures must be monitored as clinically warranted [3-4]. Once the pathogenic variant of ARID1B has been detected in an affected family member, prenatal and preimplantation genetic testing can be conducted [4].

## Conclusions

This case involving a nine-year-old female patient diagnosed with Coffin-Siris syndrome and notable hypertrichosis highlights the intricacies associated with the diagnosis of genetic disorders. It stresses the importance of maintaining a heightened level of suspicion by healthcare professionals for rare syndromes in pediatric patients who exhibit developmental delays and dysmorphic characteristics. Prompt genetic assessment can enable swift diagnosis and comprehensive care strategies. Also, genetic counseling is essential for family planning and understanding the inheritance pattern. This case report offers valuable insights into a rare syndrome; sharing these insights with physicians may aid in the earlier identification of the syndrome in the future.
